# Nitrate Uptake Affects Cell Wall Synthesis and Modeling

**DOI:** 10.3389/fpls.2017.01376

**Published:** 2017-08-08

**Authors:** Simone Landi, Sergio Esposito

**Affiliations:** Dipartimento di Biologia, Università degli Studi di Napoli Federico II, Complesso Universitario di Monte Sant'Angelo Napoli, Italy

**Keywords:** abiotic stress, *Arabidopsis*, ammonium, tomato, xyloglucane synthesis, pectin synthesis, cellulose synthesis, nitrogen assimilation

## Abstract

Nowadays, the relationship(s) about N assimilation and cell wall remodeling in plants remains generally unclear. Enzymes involved in cell wall synthesis/modification, and nitrogen transporters play a critical role in plant growth, differentiation, and response to external stimuli. In this review, a co-expression analysis of nitrate and ammonium transporters of *Arabidopsis thaliana* was performed in order to explore the functional connection of these proteins with cell-wall related enzymes. This approach highlighted a strict relationship between inorganic nitrogen transporters and cell wall formation, identifying a number of co-expressed remodeling enzymes. The enzymes involved in pectin and xyloglucan synthesis resulted particularly co-regulated together with nitrate carriers, suggesting a connection between nitrate assimilation and cell wall growth regulation. Major Facilitator Carriers, and one chloride channel, are similarly co-expressed with pectin lyase, pectinacetylesterase, and cellulose synthase. Contrarily, ammonium transporters show little or no connection with those genes involved in cell wall synthesis. Different aspects related to plant development, embryogenesis, and abiotic stress response will be discussed, given the importance in plant growth of cell wall synthesis and nitrate uptake. Intriguingly, the improvement of abiotic stress tolerance in crops concerns both these processes indicating the importance in sensing the environmental constraints and mediating a response. These evaluations could help to identify candidate genes for breeding purposes.

## Introduction

Cell wall development and remodeling are crucial processes for plants. The molecular and biochemical modifications of cell wall play critical roles in various aspects of plant physiology such as, differentiation, senescence, abscission, plant–pathogen interactions, abiotic stress response, plant growth, and others (Marowa et al., 2016). Cell wall is a necessary plant characteristic, mainly composed by polysaccharides, such as, cellulose and hemicellulose; pectins; lignin, and structural proteins (Guerriero et al., 2014, 2016). A major feature of the cell wall is its dynamic and active structure, remodeled during key stages of development, and in response to external stimuli. Therefore, during the plants life there is an incessant assembly, disassembly, and re-arrangement of the cell wall (Marowa et al., 2016). These processes are critical for plant development and acclimation, because the cell wall loosening is a direct cause of cells expansion and plant growth (Fukuda, 2014).

An interesting example is the cell wall remodeling during the stress response, by the activation of a wide range of enzymes involved in cell wall loosening (Tenhaken, 2015). This regulation represents a crucial point for tolerance to drought and salinity in crops (e.g., tomato; rice), when huge number of genes was differentially expressed upon stress (Iovieno et al., 2011; Landi et al., 2017b). Furthermore, cell wall is differently modified by biotic stress and pathogen attacks, revealing its functional plasticity (Bellincampi et al., 2014).

Among the mechanic modifications required for cell wall remodeling, the enzymes mainly involved include xiloglucan endotransglucosylase/hydrolase, expansine, enzymes involved in pectin modification (e.g., pectinesterase; pectin lyase), peroxidase (Tenhaken, 2015; Franciosini et al., 2017; Landi et al., 2017b). These enzymes are consistently regulated during nutrient deficiency (as nitrogen and/or sulfur deprivation), in order to allow the correct uptake of these elements (Fernandes et al., 2013). Particularly, N deficiency induces cell wall loosening: N is mainly assimilated in plants as nitrate (${\text{NO}}_{3}^{-}$) by specific transporters (Fan et al., 2017). This family includes a number of carriers generally described as low or high affinity transporters, playing different roles depending on the soil availability of N. In addition, plants can assimilate N as ammonium (${\text{NH}}_{4}^{+}$) by specific channels (Glass et al., 2002).

In the present study, an overview of the relationship between cell wall remodeling and nitrogen uptake will be provided. The co-expression analysis of *Arabidopsis thaliana* nitrate and ammonium transporters will be explored, in order to identify how cell wall enzymes relate to N assimilation, and clarify the concurrent processes involved in cell wall re-organization. A final survey with a perspective on the importance of N assimilation and cell wall modification upon abiotic stress will be given.

## N uptake and cell wall remodeling: a co-expression analysis

The relationships between N accumulation and plant cell wall remodeling are argument of debate. The molecular cross-interactions between these processes are still unclear: therefore, nitrogen and ammonium transporters were identified in *A. thaliana*, and co-expression analysis was made using the ATTED-II software version 8.0 at http://atted.jp (Aoki et al., 2016).

In detail, six low affinity nitrate transporters (At1g12110, At1g69850, At1g32450, At1g27080, At1g69870, At4g21680), two “major facilitator super family” proteins (At1g52190, At3g16180), seven high affinity nitrate transporters (At1g08090, At1g08100, At5g60780, At5g60770, At1g12940, At3g45060, At5g14570), and six ammonium transporters (At4g13510, At1g64780, At1g64780, At4g28700, At3g24290, At2g38290) were selected at this purpose.

The chloride channel A (*CLCA*–At5g40890) was chosen based on its capability of 2 ${\text{NO}}_{3}^{-}$/1H^+^ exchange.

It should be noted that ammonium transporter 1.3 (*AMT1.3*–At1g64780); and 1.5 (*AMT1.5*–At3g24290) showed no co-expression in the database utilized, and thus these carriers were excluded in the present analysis.

Intriguingly, several cell wall related genes are co-expressed with nitrate and ammonium transporters (Table 1). Particularly, it is worth noting the presence of a number of enzymes involved in cell wall loosening: during nitrogen assimilation, a disassembly of the cell wall could be necessary for an enhanced N uptake, allowing a correct cell and plant growth. Furthermore, this behavior suggests that a right balance of cell wall loosening and thickening is desirable during plant growth, in order to correctly supply nutrients for biosynthesis of both primary and secondary cell walls. This balance could be enhanced by adequate nitrogen assimilation.

**Table 1:** Co-expression analysis of *Arabidopsis* nitrogen and ammonium transporters, obtained using the ATTED-II database.

***A. THALIANA*** **LOW AFFINITY NITRATE TRANSPORTER**												***A. THALIANA*** **AMMONIUM TRANSPORTER**							
**At1g12110 NT 1.1.**		**At1g69850 NT 1.2**.		**At1G32450 NT 1.5**		**At1G27080 NT 1.6**		**At1g69870 NT 1.7**.		**At4g21680 NT 1.8**.		**At4g13510 AMT 1.1**		**At1g64780 AMT 1.2**		**At4g28700 AMT 1.4**		**At2g38290 AMT 2**	
**Guard cells–lateral roots**		**Roots hairs and epidermids**		**Roots pericycle cells**		**Vascular tissue of funiculus and silique**		**Phloem**		**Xylem**		**Plasma membrane**		**Endodermal and cortical cells of root**		**Plasma membrane–leaf, flower, pollen**		**Plasma membrane and cytoplasm**	
**Co-expressed genes**	**MR**	**Co-expressed genes**	**MR**	**Co-expressed genes**	**MR**	**Co-expressed genes**	**MR**	**Co-expressed genes**	**MR**	**Co-expressed genes**	**MR**	**Co-expressed genes**	**MR**	**Co-expressed genes**	**MR**	**Co-expressed genes**	**MR**	**Co-expressed genes**	**MR**
PMA2	4	FMO	4.6	HAD	1	**CESA10**	3.2	Major facilitator	1.4	TH8	3	Lipase	5	CLC-B	3.5	At5g19270	3	ERD6	5.6
NIR1	7.1	Hydrolase	8.4	PHO1	2.8	DUF821	6.9	UGT84A3	1.7	LTP	5.5	**GSR 1**	5.7	Cysteineases	4.4	Galactose oxidase	5.2	SERK3	6
NR1	7.9	Transcription	8.4	At2g28780	3.9	TLP5	7	Major facilitator	2	Rap2.6L	5.6	Kinase	9.4	Transporter	5.7	RmlC-like cupins	8.9	UGT71C5	9.4
REF1	13.2	CNGC5	17	UMAMIT18	4.9	RGP4	7.3	CAX7	3	UGT76E12	13.4	LHT1	9.9	At2g15020	11.5	At1g15830	9.8	RLK7	11.2
GSR2	16.3	**TBL40**	18.9	MYB59	5.3	ASD2	7.8	GPT2	4.2	BGLU11	13.4	PP 2C	9.9	cPT4	16.3	galactokinase	10.6	PGP21	11.4
UGT72E1	18.4	ACR3	20.4	DUF599	5.5	BAN	8.8	YSL1	5.5	**XTH11**	16.7	AMT2	14.1	At3g56290	18.8	inhibitor	10.9	AMT1;1	14.1
SULTR1;2	19.6	Plant calmodulin	22.2	Galactose mutarotase	6.3	MYB5	9.2	Protease	6.3	Nitrate transporter 2.6	19.4	HIR2	17.8	NAS1	19.4	Ubiquitin-like	16	EXO70B2	14.6
PSY1R	21.4	XIP1	22.3	UMAMIT29	6.7	UGT73C2	10.4	Transferase	6.5	Related to AP2 6	24.4	PEN3	18.5	MYC4	19.6	UPF0497	17	kinase	16.9
FMO GS-OX5	29.7	PSY1R	31	DUF716	8.1	ligase	12	MATE efflux	6.7	SRG2	24.4	PLAC8	18.7	At5g19970	21.2	AGL57	17.5	IQM1	19.8
GTR2	37.1	XLG1	35.6	MYB48	8.7	CYP709B1	12	SPSA1	9.2	GLYI7	27.6	RLK	18.9	Transferase	22.7	At1g15840	20.2	Transmembranes 14C	20.7
TIP2;2	39.4	ADR1-L1	38.2	HMA4	9.2	Major facilitator	12.7	PES1	10.8	DIN11	29.6	PMR2	22.2	myb	25.3	At2g22060	23	transferase	20.8
G6PD2	40.1	Galactose oxidase	49.1	Oxidoreductase	10.6	Major facilitator	12.7	JR2	12	DNA-binding	30.2	BIR1	23.1	**XTR8**	26.5	Glycine-rich	23.4	BIK1	21
CYP71B7	41.4	TET5	52.8	Major facilitator	10.9	**RmlC-like** **cupins**	16.3	UGT71B1	12.4	ORS1	33.7	PMT5	26.9	Transferase	29.5	Transferase	24.4	Isomerase	22.4
Chaperonin	46	**XTH27**	54.4	**Endopeptidase**	11.5	Transferase	18	MT2A	13.9	GSTU4	34.2	DUR3	29.9	FADA	29.9	Transposable	25.1	Hydrolase	23.4
Transcription	48.1	PHX21	57.5	At2g21560	11.8	TT10	20.9	LOX2	14	SRG1	36.1	Major facilitator	32.4	CAT2	30	**CSLD6**	26.1	CRK29	23.5
CA4	52	**UGE1**	57.8	UMAMIT17	12.2	MBOAT	21.9	Tetratricopeptide	14.5	Decarboxylase	46.9	**Chitinase**	33	GBSS1	30.8	IDH-III	26.5	SUC1	24.2
UPM1	55	STP4	58.7	VIT	12.4	Hydrolase	23.8	transferase	17.6	2OG	47	WR3	33.3	Glutaredoxin	34.6	CHX25	27.7	BIR1	24.6
NR2	56.4	Leucine-rich repeat	58.8	DUF599	13	OPT5	24.7	COR15A	21.2	AGP10	47	MCP1c	36.2	CAD4	35.9	GRP17	28.6	CRK28	25.6
Zinc finger	58.6	SET7/9	59	UMAMIT31	13.1	DUF579	25.7	SWEET4	21.5	NAC019	49.8	ERD6	40.1	**dirigent-like**	36.6	COPT3	31	Zinc finger	26.4
AAP5	58.7	Protein kinase	59.3	SLAH1	13.4	MES19	27.8	UGT76E11	22.6	Major facilitator	51.2	SOBIR1	40.7	ACN1	37.5	ENODL22	32.5	XBAT34	27.5
KT1	59.1	At3g52240	62.6	UMAMIT30	13.9	UMAMIT15	28.8	transporter	23.8	**XTR6**	51.9	ACA11	43.1	PME1	40.7	TIR-NBS-LRR	33.3	CNGC10	30.9
Oxidoreductase	67.5	Related to AP2 2	69.5	Major facilitator	14.5	**Pectinacetylesterase**	30	NAT2	28.4	NAC3	54.2	Protease	44.7	PRH43	41	At3g44140	34.5	At2g18690	31.1
TBL27	69.2	NPC1	70.3	AAP2	14.9	MBOAT	30.3	**GDSL Hydolase**	29.9	Rossmann-fold	54.5	EXO70B2	45.3	SPS2	41.8	Glycine-rich	35.1	**FAD binding** **Berberine**	32.5
LEA	71.6	PMIT1	70.4	Glycine-rich	14.9	SHP2	30.9	MATE efflux	30.4	AKR4C8	55.3	ALA1	46.6	NCS1	42	UGT84B2	36.9	PLAC8	34.6
Transporter	72.8	Duplicated homeodomain	72.4	Transporter	15	Rossmann-fold	31.8	ZHD10	33.3	ILR1	57.9	STP4	47.2	At5g43150	42.1	At2g18115	37.4	WCOR413	37.5
UGT84A4	75.9	DUF946	73.2	At4g34600	15	Inhibitor	32.8	PSK5	35.4	Transferase	66.5	Kinase	52.6	COPT2	42.6	DUF220	40	Kinase	38.6
Transferase	76.2	Fragile-X-F-associated	77.1	DNA-binding	15.2	IPT6	34.2	Major facilitator	35.8	CAD1	67.4	IQM1	53.1	PSY1R	45.2	Transposable	41.4	SYR1	39.1
EFE	82.8	At2g17710	77.5	UMAMIT28	15.3	MES4	34.4	CCT motif	36.4	Oxidoreductase	71.7	CRK19	53.2	Major facilitator	47.2	Plant self-incompatibility	41.4	MATE efflux	40.1
**HAD**	85.5	SEC14 cytosolic factor	80.5	UMAMIT20	16	TT12	36.5	RLP33	37.6	BT4	72.8	SERK3	53.6	SIGE	48	Major facilitator	45.2	At4g25030	40.1
**CSY4**	88.4	**GASA1**	86.4	UMAMIT11	17.8	**Peroxidase**	37.1	NAC019	38.8	**PRX52**	73.5	Kinase	53.8	ADT6	48.1	VIT	45.5	PLAC8	42.4
**MAJOR FACILITATOR SUPER FAMILY**				***A. THALIANA*** **HIGH AFFINITY NITRATE TRANSPORTER**														**Chloride Channel**	
**At1g52190 NT 1.11**		**At3g16180 NT 1.12**		**At1g08090 NT 2.1**.		**At1g08100 NT 2.2**.		**At5g60780 NT 2.3**		**At5g60770 NT 2.4**		**At1g12940 NT 2.5**		**At3g45060 NT 2.6**		**At5g14570 NT 2.7**		**At5g40890 CLCA**	
**Plasma membrane—leaf phloem**		**Plasma membrane—leaf phloem**		**Plasma membrane—root, shoot**		**Plasma membrane**		**plasma membrane–shoot apex, vascular leaf**		**Plasma membrane**		**Guard cells–Inflorescence–stem**		**Chloroplast–flower, guard cells, root**		**Tonoplast–reproductive organs and seeds**		**Cellular and vacuolar membrane**	
**Co-expressed genes**	**MR**	**Co-expressed genes**	**MR**	**Co-expressed genes**	**MR**	**Co-expressed genes**	**MR**	**Co-expressed genes**	**MR**	**Co-expressed genes**	**MR**	**Co-expressed genes**	**MR**	**Co-expressed genes**	**MR**	**Co-expressed genes**	**MR**	**Co-expressed genes**	**MR**
**Pectin lyase-like**	1.7	**TUB5**	4.2	PP2C	1	Nitrate transporter 2.4	1.4	At5g38320	2	PP2C	6	GLN1;4	1	Nitrate transporter 2.3	3.5	GDSL-like Lipase	6.6	VAC-INV	1.4
IAA7	2.2	WLIM2a	9.2	Oxygenase	3.2	PP2C	2.5	Nitrate transporter 2.3	3.5	Hydroxylase	14	Thioredoxin	15.2	DNA-binding	6.9	AER	8.7	At1g49500	1.4
Domain	3.3	TUB1	9.6	HPP	6	MBD3	2.5	Inhibitor	3.5	Cysteine/ Histidine-rich	22.4	YSL7	20.9	Inhibitor	15.3	At5g64230	11.6	Hydrolase	6.3
Glycosylase	3.5	DUF1645	9.8	RWP-RK	6.3	Oxygenase	3.3	PRB1	4.2	GNS1/SUR4 membrane	26.5	FRK1	26	Nitrate transporter 1.8	19.4	Heavy metal detox	13.9	TIP2	6.7
**Pectin lyase-like**	4.2	DRT100	10.7	TIR-NBS-LRR	6.9	NRT2;1AT	7.1	LMI1	39.1	**CSLB02**	28.2	CAT1	26.5	WRKY28	24.9	G3Pp4	15.2	PIP1A	8.5
LUP1	4.4	PGP19	12.7	GSTF14	12.4	HPP	13.2	ASML2	41.7	CYP702A2	36.9	Cysteine/Histidine-rich	27.8	DUF642	37.4	RCC1	15.5	PIRL4	8.5
PKS2	4.6	Transferase	15.2	WR3	13.8	RWP-RK	21.9	SUC6	44.5	MBOAT	38.5	CAT5	40.6	LTP	38.2	Transporter	18.4	Beta-xylosidase 1	9.4
PIN7	4.9	ERD3	15.4	NAS2	18	TIR-NBS-LRR	54.7	Transcription	52.6	Mannose-binding lectin	40.6	Transporter	47.8	SHB1	53	GolS3	21.6	HAD	10
P1R1	5.7	Transferase	15.9	PP2-A3	19.3	Transferase	60.2	NUB	70.6	CYP96A14P	45.1	NAC048	53.8	MLO12	57.5	Nitrate transporter 1.7	23.8	SPF1	10
BEE2	6	DNA-binding	17.9	At5g10210	20.4	LEA3	62.7	**Peroxidase**	80.9	Terpenoid synthases	49.6	ZIP5	55.6	SLAH2	65.7	At1g68500	24.2	PATELLIN1	12.1
**DGR2**	6.9	Glycosylase	18.8	Kinase	28.6	Transposable	72.2	Transferase	86.5	DC1	55.7	CHX16	59.7	CAT1	70.7	At3g19920	31.8	phosphoesterase	14.4
TCP15	7.3	**Pectin lyase-like**	18.9	TIR-NBS-LRR	34.6	GSTU21	100.5	DNA-binding	102.1	WSD1-like	57	Inhibitor	63.9	Zinc finger	73	DNA bromodomain	41.6	beta glucosidase 16	15
At1g67050	7.6	Kinase	20.7	**Pectin lyase-like**	37.1	Glutamate receptor	101.5	PP2C	104.9	TLC	67.7	RLP21	78.2	WRKY8	76.8	**Glycosyl** **hydrolase**	42.7	TauE/SafE	15.4
DWF3	7.8	LYK3	21.9	Glutamate receptor	37.2	At1g49260	103.4	LEA	120.1	Terpenoid synthases	69.5	OPT1	78.4	YSL7	77.6	chaperonin	44.9	beta galactosidase	17.2
**DUF642**	9.5	TRM2	22.2	Major facilitator	46.9	DNA-binding	105.7	RPP27	123.9	DNA-binding	90.7	Thioredoxin	79	Kinase	79.3	UDP-Glycosyltransf	46.6	TMP-A	18
GASA6	9.9	Major facilitator	24.2	Kinase	48.4	DNA-binding	116.8	UMAMIT32	133.1	Transporter	94.5	DNA-binding	79.3	Cysteine/ Histidine domain	81.8	UGT76E11	54.4	Phosphorylase	21
PRA1.F1	11.5	**Pectinacetylesterase**	24.3	Protease	48.8	Cysteine/ Histidine-rich	118.5	HDG4	151.6	Cysteine/ Histidine-rich	101.2	RWP-RK	80.7	transporter	94.4	GIA1	60.7	PIP1D	22.2
WAV5	12	RPT3	25.1	RING/U-box	49.5	At4g16090	127.1	F-box	154.6	Oxidoreductase	105.7	Kinase	87.7	Major facilitator	94.9	CYP72A15	64.7	Major Facilitator	23.4
**TIP2;1**	12.2	Gibberellin-regulated	26.8	Kinase	53.5	Transposable	128.1	Transposable	173.5	RWP-RK	119.8	CRK24	90.3	WR3	96.6	LKP2	65.6	**Pectin-lyase like**	25.4
Phosphoesterase	12.4	PLA2-ALPHA	27.5	PGM	55.5	At2g18610	129.1	F-box	188.1	Cysteine/ Histidine-rich	125.7	MCP1c	92.8	SAUR-like auxin-responsive	96.8	LEA	68.3	PIP2A	25.4
**Pectin lyase-like**	14	**At3g52500**	27.8	**Peroxidase**	57.8	At3g50250	130.2	At5g48200	200.8	At1g07680	136	ACR6	96.3	2OG	97.6	TLC	69.2	Major Facilitator	26.3
**EXPA11**	14.5	Homeodomain-like	29.1	Ca-dep lipid-binding	60.3	Kinase	132.5	Transposable	205	**Peroxidase**	136.2	**Bifunctional** **inhibitor**	104.1	Kinase	99.8	DNase	69.3	**CSLA3**	26.5
PIN4	14.6	**FRUCT5**	29.6	TAC1	60.7	PUP15	139.5	At5g28800	211.6	Cysteine/ Histidine-rich	138.1	At1g51920	123.5	Transposable	111.1	COR15B	71.5	ZYK4	26.8
Phosphodiesterases	14.7	GRH1	31.4	Kinase	60.8	At1g53640	141	At4g16090	211.8	Transposable	140.8	PTR3	128.2	MYB2	124.1	SOM	76.7	ATRR4	27
DUF617	16.4	**PME3**	33.9	TIR-NBS	61.5	Kinase	157	At4g11930	223.8	PLAC8	144.3	zinc finger	132.4	**SS3**	124.8	RLP33	77.5	**Pectin-lyase like**	29.8
**EXPA8**	16.9	TUB6	35.1	SAUR-like	64.5	C2	161.5	Transferase	235.6	Cysteine/ Histidine-rich	145	lectin receptor kinase	132.8	Nitrate transporter 2.1	125.4	CHY2	81.2	PSY1-R	29.9
**Pectin lyase-like**	17.7	TET7	36.3	G6PD3	71.3	At3g44140	173.9	Galactose oxidase	237.3	PEN2	146.2	Lipase class 3	135.7	Kinase	133.7	Na/Ca exchanger	84.3	TMK-1	30.2
TCP11	19.8	TBR	37.4	GSTU21	76.8	LEA	180.1	RING/U-box	244.9	Cysteine/ Histidine-rich	146.2	DUF1218	138	Kinase	136.2	RD29A	87.5	TIP1:2	30.7
Glycosylase	20.4	Kinase	39.5	Kinase	77.1	Kinase	183.1	Transposable	247	CSLB01	146.6	RLK6	139.8	ELI3-2	139.7	**At1g21670**	90.1	At3g27390	33
**EXP3**	20.8	**XTH4**	42.7	Cysteine/ Histidine-rich	79.5	**Pectin** **lyase-like**	256.4	Separase	249.5	PRA1.G1	149.8	SHB1	139.9	**Plant invertase**	202.1	**Glycosyl hydrolase**	108.5	SnRK3.17	33

*The co-expression degree was estimated as Mutual Rank (MR), as described by Aoki et al. (2016), and shown on the right side of each column. Cell wall related genes (yellow highlighting genes) were identified by Gene Onthology categories*.

*The identification of the main interesting cell wall related genes is as follow: At3g52500 (Eukaryotic aspartyl protease); Bifunctional inhibitor (Bifunctional inhibitor/lipid-transfer protein/Seed storage 25 albumin protein); CESA and CSLB (Cellulose synthase); CSY (Citrate synthase); DGR2 (Protein with unknown function); DUF (Protein with unknown function); Dirigent (Disease resistance-responsive dirigent like protein); Endopeptidase (Substilin-like serine endopeptidase family protein); EXPA (Expansin); EXP3 (Barwin like endoglucanase protein); FRUCT5 (Beta-fructofuranosidase 5); GASA (GAST1 protein homolog); GDSL hydrolase (GDSL-like Lipase/Acryhydrolase protein); GSR (Glutamine syntethase); HAD (HAD superfamily, subfamily IIIB acid phosphatase); Plant invertase (Plant invertase/pectin methylesterase inhibitor superfamily); PME (Pectin methylesterase 3); PRX (Peroxidase); SS3 (Strictosidine synthase 3); TBL (Protein with unknown function); TIP2:1 (Tonoplast intrinsic protein); TUB5 (tubulin beta-5 chain); UGE (UDP-D-glucose/UDP-D-galactose 4-epimerase 1); XTR (Xyloglucan endo-transglycosylase); XTH (xyloglucan-endotransglucosylases/hydrolases)*.

Consistent with these considerations, Fernandes et al. (2016) showed a diversified molecular expression of the cell wall loosening related genes in *Vitis viniferae* callus subjected to nitrogen, sulfur, and phosphorus deficiency, highlighting that N affects the cell wall responses more severely than other nutrients.

As shown in Table 1, low affinity and high affinity nitrate transporters showed similar number and type of cell wall related co-expressed genes. Otherwise, ammonium transporters showed a lower co-expression with cell wall related genes; this would probably suggest minor, or absent relationship(s) with cell wall remodeling.

Examples of cell wall remodeling genes which appear related to nitrogen transport are pectinase, involved in pectin degradation, such pectin lyase (At4g23820, At3g07010, At3g16850, At5g48900, At5g14650, At3g57790, At3g16850), pectinacetylesterase (At1g09550, At5g23870), or pectin methylesterase (At3g14310). Particularly, the cleavage of homogalacturonans by pectinesterases produces substrates for polygalacturonase and pectin lyase, acting in the cleavage of the polygalacturonic acid (Sun and Nocker, 2010).

These genes are important members of fruits' maturation network (Marín-Rodríguez et al., 2002), and previous studies described their involvement in the abiotic stress response (Hong et al., 2010; Tenhaken, 2015; Landi et al., 2017b). It has been proposed that pectins are able to form gel structures that increase cell wall consistency (Fernandes et al., 2016).

The activation of pectinase(s) together with nitrogen transporters could induce the relaxation of the cell wall.

Other important actions associated with nitrogen uptake are the modification of xyloglucans. A number of enzymes involved in this process were co-expressed with nitrate transporter such xyloglucan-endotransglucosylases/hydrolases (*XTH*—e.g., At3g44990, At3g48580, At2g06850), xyloglucan-endo/transglycosilase (*XTR*—e.g., At4g25810), and expansins (e.g., At1g20190–At2g40610). Xyloglucans are the major hemicellulosic polymers of dicot plants, playing a critical role in cellulose fibrils connection. Modification in their content is an important process regulating several physiological plant responses by the cell wall remodeling (Tenhaken, 2015; Marowa et al., 2016). It was proposed that xyloglucan regulation by expansins could improve the efficiency of nutrient uptake. In fact, several types of expansins respond to different nutrient deficiencies including nitrogen, phosphorus, potassium, and iron ones (Li et al., 2014).

Furthermore, expansins have been proved to play a pivotal role in several aspects such fruit ripening and softening, abiotic stress tolerance, and crops yield (Zhou et al., 2014; Minoia et al., 2015; Marowa et al., 2016).

Interestingly, the major facilitator superfamily genes At1g52190–*AtNT 1.11* and At3g16180–*AtNT1.12* are consistently co-expressed together with several cell wall relaxation genes; it must be underlined that these transporters play an important role in plant physiology translocating nitrate from phloem to xylem.

Particularly, their action appears critical for high-nitrate-enhanced shoot growth, and for nitrate translocation from old to young leaves. These processes represent key points affecting biomass production, and crop yield (Hsu and Tsay, 2013).

Finally, nitrate transporter and cell wall related processes are connected also during embryogenesis. The *AtNRT1.6* is expressed in reproductive tissues, namely vascular tissue of the silique and funiculus. This transporter plays a critical role during early embryogenesis phase (Almagro et al., 2008): interestingly, this gene was co-expressed with cellulose synthase A (*CESA*–At2g25540). Previous studies reported that several members of this family are necessary for a correct embryogenesis (Beeckman et al., 2002; Goubet et al., 2003). This evidence corroborated the idea of a strict connection between nitrogen uptake and cell wall regulation in various aspects of plant development and morphogenesis.

## The relationship between nitrogen transporter and cell wall upon abiotic stress

It is worth to point out that both nitrate transporters and cell wall remodeling enzymes play crucial roles in response to various abiotic stresses (Tenhaken, 2015; Fernandes et al., 2016; Fan et al., 2017; Landi et al., 2017b).

Among nitrate transporters, *AtNRT1.1* (At1g12110) was identified as a salt and drought stress responsive gene (Guo et al., 2003; Álvarez-Aragón and Rodríguez-Navarro, 2017). This gene is expressed in guard cells and plays an important role in stomata opening: *AtNRT1.1*. mutants showed an enhanced drought tolerance (Guo et al., 2003).

Further, *AtNRT.1.1* plays a major role in Na^+^ and Cl^−^ assimilation in both normal and high salinity conditions, suggesting its role in salt stress tolerance (Álvarez-Aragón and Rodríguez-Navarro, 2017). Interestingly, co-expression analysis showed this gene less co-expressed with cell wall related genes (Table 1): this confirms that cell wall remodeling genes were diversely down-regulated during abiotic stress in order to limit the damage (Leucci et al., 2008). Intriguingly, *AtNRT1.1*. showed a number of stress-related coexpressed genes such as, tonoplast intrinsic protein (*TIPs*–At4g17340), glucose-6P dehydrogenase (*G6PDH*–At5g13110), heat shock proteins (*HSP*–At5g02480), late embryogenesis proteins (*LEA*–At3g52470; Boursiac et al., 2005; Ma et al., 2006; Basile et al., 2011; Esposito, 2016; Landi et al., 2017a), thus highlighting its role in abiotic stress response (Table 1).

Another interesting nitrate transporter involved in abiotic stress response is *AtNRT1.8* (At4g21680): cadmium (Cd^++^) stress strongly stimulated the accumulation of this transporter in roots, and *A. thaliana* plants with mutated *AtNRT1.8* showed increased sensibility to Cd^++^ stress (Gojon and Gaymard, 2010). Intriguingly, as showed in Table 1, *AtNRT1.8* is co-expressed with a number of cell wall related genes, namely *XTH11* (xyloglucan-endotransglucosylases/hydrolases), *XTR6* (xyloglucan-endo/transglycosilase), and *PRX52* (peroxidase superfamily). Particularly, peroxidase activity was assisted by a number of antioxidant enzymes such as, glutathione S-transferase (*GSTU4*), NAD(P)-linked oxidoreductase (*AKR4C8*), and others (Table 1). This could be necessary to regulate the increased of reactive oxygen species (e.g., H_2_O_2_), enhancing the mechanical stability of the cell wall, and thus stress tolerance (Tenhaken, 2015).

Further, *CLCA* (At5g40890) is a chloride channel that plays a role as ${\text{NO}}_{3}^{-}$/H^+^ exchanger, useful to accumulate nitrate in vacuoles (De Angeli et al., 2006). Recently, this transporter was reported as related to *PP2A-C5* (At1g69960) during salt stress response (Hu et al., 2017); the co-expression analysis showed a relationship with cell wall related proteins such as, pectin lyase (At3g57790 and At3g16850); cellulose synthase C; and with aquaporines such *TIPs* (tonoplast intrinsic proteins) and *PIPs* (Plasma membrane intrinsic proteins). The co-expression of *TIP2* (At3g26520) and *TIP2.1* (At3g16240) confirms the critical role of *CLCA* in nitrate translocation into the vacuoles as well. Interestingly, *NTR1.1* is co-expressed with tonoplast intrinsic protein *TIP2.2* (At4g17340). Particularly, nitrate allocation from/to vacuoles suggested a central role during plant adaption in N-rich and N-deficient environments (Fan et al., 2017). Recent evidence indicated the role of phosphatidylinositol-3,5-bisphosphate as signal for nitrate translocation in vacuoles by the activation of *CLCA* (Carpaneto et al., 2017).

Further, the regulation of the nitrate allocation into the vacuoles was assisted by peptide transporters (*PTRs*), such as, *AtPTR4* (At2g02020) and *AtPTR6* (At1g62200); these proteins showed vacuole specific localization, thus playing a role in nitrate storage in the plant cell (Weichert et al., 2012). Fan et al. (2017) reported that *NRT2.1* plays an important role in resistance to drought. This action was reported in different species such as, *Arabidopsis* and *Brassica*, together with *NRT1.1* and *NRT1.5* (Goel and Singh, 2015; Fan et al., 2017). Other authors reported that *NRT2.1* regulated root hydraulic conductivity, by altering ${\text{NO}}_{3}^{-}$ accumulation (Li et al., 2016). Furthermore, this nitrate transporter positively regulates the translational levels of *PIPs*; the bioinformatic analysis highlights the co-expression of this transporter with cell wall related genes, such pectin lyase and peroxidase; and with abiotic stress related genes such protein phosphatase 2C (*PP2C*), glutathione S-transferase (*GST*), *G6PDH*, and others, thus confirming that nitrogen transporters, cell wall remodeling enzymes, and others genes together contributes for abiotic stress tolerance.

## Transcriptomic modification in adverse environment: nitrate and cell wall candidates genes for tolerance in crops

Nowadays, next generation sequencing (NGS) provides for new insight into crops genetic breeding, generating huge amount of data, mapping across crops population, and discovering useful genes, QTL and genomic traits (Cobb et al., 2013).

The improvement of tolerance in crops vs. abiotic stress remains today an important focus for plant biology researchers because this reduces plant growth, development, and productivity (Reynolds and Tuberosa, 2008; Cardi et al., 2015; Ruggiero et al., 2017). This promising strategy can be prosecuted by applying modern molecular and -omics techniques, together with the study and the analysis of traditional landraces (Van Oosten et al., 2016; Landi et al., 2017a,b). In the last years, many researchers investigated this topic using NGS; in tomato (*Solanum lycopersicum*), 966 differential expressed genes (DEGs) have been identified upon drought; among these, at least 50 genes involved in cell wall remodeling and nitrate transport were identified. Particularly, 20 clusters of genes were grouped, and their transcripts show similar expression trends (Iovieno et al., 2011).

Some clusters showed interesting correlations: in cluster 4, expansin (Solyc06g049050), nitrate transporter (Solyc12g006050), cellulose synthase (Solyc04g071650), and *XTH* (Solyc02g091920); in cluster 5, cellulose synthase (Solyc04g077470), expansin (Solyc02g088100), nitrate transporter (Solyc03g113250), and *XTH* (Solyc07g052980).

Similarly to other abiotic stress, nutrient deprivation negatively influences crops yield. Nitrogen deficiency is a critical cause of yield loss, but N fertilizer consumption has become one of the major costs of crop production (Zhao et al., 2015).

A huge transcriptomic modification in durum wheat (*Triticum turgidum*) upon nitrogen starvation highlighted 4,626 DEGs in different organs such as, roots, leaves, stems, and spikes (Curci et al., 2017). An interesting enrichment of GO categories related to “Cell Wall Biogenesis” and “Cellulose metabolism” in leaves was reported, highlighting the relationship between nitrogen nutrition and regulation of the integrity of cell wall. Also, a number of up-regulated high affinity nitrate transporters in root and flag leaf (e.g., *NT2.3* and *NT2.5*) were found, while numerous cell wall related genes showing a transcriptional regulation induced by nitrogen starvation. Examples of these are pectin lyase, expansin, and wall associated kinase (*WAK*). Particularly, *WAKs* play critical roles in root growth under N limitation (Kiba and Krapp, 2016). Intriguingly, the correlation among *WAKs* and nitrogen deficiency was also observed in two lines of Tibetan barley *(Hordeum vulgare)* expressing nitrogen transporter with genomic variants (Quan et al., 2016).

Moreover, nitrogen starvation was studied in rice (*Oryza sativa*; Yang et al., 2015). This stress induced the modification in the expression of 1,158 genes in leaves, and 492 in roots. Part of these were identified as cell wall related genes: in roots it has been reported the expression of few genes involved in cell wall degradation, such fasciclin-like arabinogalactan protein (Os10t0524300), and sulfated surface glycoprotein (Os10t0524300). On the contrary, in leaves a higher number of DEGs related to various aspects of cell wall regulation was reported, such fasciclin-like arabinogalactan protein (Os01t0668100), beta-galactosidase (Os06t0573600), UDP-glucuronic acid decarboxylase (Os03t0278000), and expansin (Os10t0555900, Os10t0556100).

Recently, Zhao et al. (2015) reported interesting results about the response of cucumber (*Cucumis sativus*) at early nitrogen shortage. Among the top enriched GO categories, the presence of genes encoding for proteins and enzymes involved in xyloglucan transferase activity were reported, underlining their role(s) in cell wall synthesis and remodeling. Further, a number of genes involved in cell wall loosening, cell expansion or cell wall component synthesis, including pectin lyases (Csa1G049960), *XTH* (Csa1G188680), pectinesterases (Csa7G447990; Csa7G343850), and expansin (Csa5G517210) were grouped in different expression clusters, and regulated during the early stage of N deficiency response. Thus, pectins breakdown under N deficiency would provide substrates to other biological processes, compensating for the depressed photosynthetic carbon assimilation. In addition, a connection between cell wall degradation and ascorbic acid metabolism can be hypothesized, in order to provide an improvement of fruit quality upon N deficiency (Zhao et al., 2015).

Interestingly, cell wall related and nitrate transporter genes interact also during heavy metal stress such as, aluminum excess (Li et al., 2017). It has been reported a critical role for the *STOP1/ART1*, a zinc finger transcription factor, which induced the expression of a number of genes related to the aluminum toxicity tolerance in crops (Yamaji et al., 2009).

**Figure 1:**
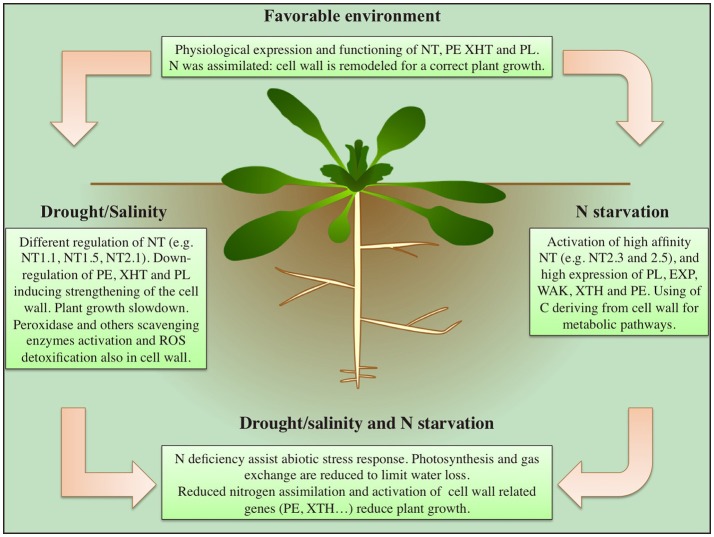
Main effects induced by drought, salinity and nitrogen starvation on nitrogen assimilation and cell wall remodeling in plants.

The effectors of *STOP1/ART1* suggest a correlation in tea plants (*Camelia sinensis*) among cell wall related enzymes (e.g., expansine and polygalacturonase); membrane proteins (e.g., magnesium transporter, UDP-glucosyl transferase, and potassium transporter); detoxification proteins (e.g., Heat shock protein 20) and nitrate transporters. Therefore, a major role in the aluminum allocation for tolerance, or accumulation, has been proposed for this protein network (Li et al., 2017). A schematic summary, describing the key events during drought, salt and N starvation responses, and their relationships between nitrogen uptake and cell wall remodeling, is proposed in Figure 1.

## Conclusions

This review provided for an updated survey between the correlation of nitrogen assimilation and cell wall related genes. These genes contribute together in several aspects of plant growth, physiology, and response to external stimuli. Evidences here described strongly support the notion of an involvement of *NT* and cell wall remodeling genes (e.g., pectin lyase, *XTH*, expansin) as a part of complex machinery involved in abiotic stress response in crops.

Further, cell wall related genes play a role in N starvation inducing cell wall relaxation and helping N assimilation. Therefore, these gene families could represent promising traits for genetic improvement in abiotic stress tolerance.

## Author contributions

SL and SE conceived the idea and wrote the manuscript.

### Conflict of interest statement

The authors declare that the research was conducted in the absence of any commercial or financial relationships that could be construed as a potential conflict of interest.
